# O004. Refractory chronic cluster headache responding absolutely to indomethacin

**DOI:** 10.1186/1129-2377-16-S1-A96

**Published:** 2015-09-28

**Authors:** Carlo Lisotto, Federico Mainardi, Ferdinando Maggioni, Giorgio Zanchin

**Affiliations:** Headache Centre, Department of Neurosciences, University of Padua, Padua, Italy; Headache Centre, Hospital of Venice, Venice, Italy

## Background

Cluster Headache (CH) attacks typically respond to triptans, in particular to subcutaneous sumatriptan and/or to inhaled oxygen. These treatments are effective in most cases. Anecdotal evidence suggests that some CH patients may respond absolutely to indomethacin, as do patients with paroxysmal hemicrania (PH) and hemicrania continua (HC). We report the case of a male with refractory chronic CH, who responded both acutely and prophylactically to indomethacin.

## Materials and methods

Following the International Classification of Headache Disorders (ICHD-3 beta) criteria, we diagnosed chronic CH in a 72-year-old male, whose headaches started when he was 56. For the first 4 years CH was episodic and later became chronic, with short remission periods lasting less than 1 month. The pain was strictly left-sided and was accompanied by conjunctival injection, lacrimation and nasal congestion. During most of the attacks the patient felt restless and had pacing activity. The mean frequency of headaches was two per day, one often occurring at night during sleep, and their duration was about 20-30 minutes. MRI and Angio-MRI of the brain were within normal limits.

## Results

The patient's attacks did not respond to subcutaneous sumatripan and oxygen inhalation. Interestingly, the patient noticed that his headaches would promptly and completely subside by injecting 50 mg intramuscular indomethacin. The patient was previously treated prophylactically with verapamil, prednisone and lithium, reporting no benefit. Given the absolute response to acute parenteral indomethacin, the patient was commenced with preventive oral indomethacin at the dose of 50 mg, three times daily. He showed a complete response in 10 days after the start of treatment; a tapering of the dose consistently led to the relapse of headaches.

## Conclusions

The clinical features and, to a certain extent, also the pathophysiology of both PH and HC, which respond in an absolute way to indomethacin, considerably overlap with those of CH. Indomethacin is largely considered to be ineffective in patients with CH, but a response to this drug is not contrary to the diagnostic criteria for CH[1]. Our case fulfils the ICHD-3 beta criteria for both chronic CH and also for probable chronic PH, lacking one criterion, i.e. frequency of attacks. The review of the literature suggests that indomethacin-responsive CH exists and that some cases may be misdiagnosed when one relies on therapeutic responsiveness to make a diagnosis. This seems to be particularly true for chronic CH, whose clinical characteristics may overlap with those of chronic PH[2].

Written informed consent to publication was obtained from the patient(s).
